# Gradient-Based Efficient Optimization Method for Surface Sensor Arrays in Microseismic Source Mechanism Inversion

**DOI:** 10.3390/s26175435

**Published:** 2026-08-27

**Authors:** Yue Kong, Ye Yuan

**Affiliations:** School of Naval Architecture & Ocean Engineering, Jiangsu University of Science and Technology, Zhenjiang 212100, China

**Keywords:** microseismic monitoring, moment tensor inversion, sensor placement optimization, condition number, analytical gradient

## Abstract

The accuracy of microseismic moment tensor inversion critically depends on the spatial configuration of surface sensor arrays, quantified by the condition number κ(G) of the dynamic response matrix. Existing optimization methods suffer from slow convergence and unreliability due to the fundamental lack of gradient information. In this specific context, the analytical gradient of κ(G) with respect to sensor positions via singular value perturbation theory and the chain rule of direction cosines are derived for the first time in this paper. Based on the analytical gradient, three optimization strategies are established: a BFGS quasi-Newton method with quadratic penalty (BP) for smoothly constrained regions; a sequential quadratic programming method (FMC/SQP) achieving the lowest condition numbers across all shapes with approximately 5000 evaluations; and a center-boundary parameterization method (CB) that exploits the structural regularity of optimal configurations to reduce variables from 2N to N−1, requiring only approximately 300 evaluations (1/16 of FMC). Systematic experiments on five region shapes and two complex constraint scenarios demonstrate that gradient methods achieve a 15–30% reduction in κ across 4 of 5 shapes and a 5–31-fold improvement in robustness, with FMC reducing the average condition number by 25% at 15.4-fold better robustness than the baseline. Synthetic inversion experiments confirm that reducing κ from approximately $1.5\times 10^{4}$ to approximately 50 reduces moment tensor inversion error by approximately 50-fold. The analytical gradient framework is transferable to sensor placement problems across multiple disciplines, and the three-method toolchain offers a graduated accuracy–efficiency trade-off for practical deployment optimization.

## 1. Introduction

Microseismic monitoring is an essential tool for characterizing hydraulic fracturing operations in unconventional oil and gas reservoirs [1,2,3], with applications expanding rapidly to geothermal development, CO_2_ geological sequestration, and mine safety monitoring [4,5,6]. The spatial distribution of induced microseismic events provides critical information about fracture geometry, stimulated reservoir volume, and stimulation effectiveness. Beyond event location, moment tensor inversion of microseismic waveforms can reveal the underlying failure mechanism—whether an event is tensile opening, shear slip, or mixed-mode failure [7,8,9], which has direct implications for optimizing fracturing design and assessing induced seismicity risk [10].

The accuracy of moment tensor inversion depends on two factors: the signal-to-noise ratio of the recorded waveforms [11] and the geometric configuration of the sensor array relative to the source [12,13]. This relationship has an extensive theoretical foundation in geophysical experimental design. For the moment tensor tests, sensor arrays were commonly determined based on experience, which should provide good coverage of the source [14,15,16]. To assess inversion accuracy, researchers conducted various studies to test the performance of different sensor arrays. Curtis [17] systematically expounded, from the perspective of model-driven Bayesian experimental design, the controlling role of observation system geometry on inversion uncertainty; Maurer, Curtis, and Boerner [18] summarized recent advances in optimizing geophysical survey design across electromagnetic, seismic tomography, and microseismic monitoring approaches. The core conclusion is consistent: under the linear forward model and Gaussian noise assumption, the condition number κ(G) of the dynamic response matrix $G\in R^{N\times 6}$ directly determines the worst-case upper bound of posterior uncertainty. Eaton and Forouhideh [19] demonstrated that sensor array solid-angle coverage controls moment tensor resolvability, explicitly showing that under certain layouts, inseparable coupling exists among different moment tensor components. Vavrycuk [20] analyzed geometric constraints on moment tensor retrieval from borehole data, and the early work of Nolen-Hoeksema and Ruff [21] established the methodological foundation for analyzing relationships between receiver geometry and source mechanism inversion quality. Ren [22] investigated the effects of sensor number and spacing in a regular grid on the inversion of shear–tensile microearthquake fracturing mechanisms, with the optimal array parameters only briefly summarized. A similar study was also carried out by Eyre and Van Der Baan [23]. These studies collectively established the decisive role of sensor geometry but did not formulate sensor placement as a systematic mathematical optimization problem.

Kong et al. [24] first concretized these principles into an operational optimization problem: minimize κ(G) with respect to sensor positions subject to region constraints, where $G\in R^{N\times 6}$ maps the six independent moment tensor components to the P-wave amplitudes observed by N surface sensors. Their numerical experiments revealed that optimal sensors tend to concentrate on the region boundary and confirmed that κ reduction directly corresponds to decreased non-double-couple components in the inverted moment tensor. Their coordinate descent method (OLD), based on random-direction polar-coordinate perturbations, can find reasonable solutions on simple regions ($\bar{\kappa }\approx 47.5$ on a circle), but exhibits four fundamental structural deficiencies: (1) fixed-direction coordinate descent—only one sensor is moved at a time, selected among four preset polar directions that are typically nearly orthogonal to the true gradient, leading to inefficient zigzag search trajectories; (2) greedy single-sensor updates—the 2N-dimensional coupled problem is decomposed into N independent subproblems, making coordinated sensor movements impossible, whereas such coordination is often key to escaping saddle points and shallow local optima; (3) extremely low information utilization—each 4N function evaluation per iteration only returns a scalar condition number without extracting any directional derivative information; (4) heuristic step-size control—near convergence, the step size is reduced by a fixed ratio (golden ratio 0.618) without gradient-based convergence assessment, preventing the optimizer from recognizing whether the neighborhood of the optimum has been reached. Consequently, OLD requires approximately 100 independent runs with only the top 75% retained for reliable results [24], and its standard deviation reaches $\sigma_{\kappa }=34.9$ on triangular regions (mean 110.7).

Beyond microseismic monitoring, optimal sensor placement (OSP) has been studied extensively in structural health monitoring and related fields. Recent reviews [25,26] systematically categorize OSP criteria (e.g., modal assurance criterion, Fisher information-based methods) and optimization algorithms (from random search to metaheuristics and learning-based methods). Representative recent work optimizes sensor layouts via Fisher-information-based damage-detectability criteria [27] and via information entropy and sensor cost trade-offs under convex relaxation [28], while other work places rupture-detection sensors on pipeline networks using an Adam-mutated genetic algorithm [29]. Within geophysics, the closest works to ours optimize the size, density, and spacing of surface grid arrays for shear–tensile moment tensor retrieval [22] and perform full moment tensor inversion with uncertainty quantification on large-N nodal arrays [30]. Our contribution differs in kind: rather than comparing a handful of hand-designed layouts, we formulate sensor placement as a continuous optimization problem equipped with an analytical gradient, thereby connecting the microseismic problem to the broader OSP literature through a unified gradient-based methodology.

The common bottleneck across all existing methods is the complete absence of gradient information. Gradient-free zero-order methods expend large numbers of function evaluations on characterizing the local function landscape through sampling, yet fail to convert this sampling information into search directions—which is precisely the role that gradient information naturally fulfills. To address this bottleneck, we derive the analytical gradient of κ(G) with respect to sensor positions by combining singular value perturbation theory with the chain rule of direction cosines from the moment tensor forward model. Based on this gradient, we establish three constrained optimization strategies—BP (penalty function), FMC/SQP (explicit constraints), and CB (constraint elimination via parameterization)—and systematically evaluate them against baseline methods across multiple region shapes and constraint scenarios.

The main contributions of this work are as follows: (1) We derive, for the first time in the sensor-placement context, the analytical gradient of the condition number κ(***G***) with respect to sensor positions. The derivation combines singular-value perturbation theory with the chain rule of direction cosines, at an overhead below 5% of the singular-value decomposition (SVD) already required for condition-number evaluation. (2) We establish three complementary gradient-based strategies, namely BP (penalty function), FMC/SQP (explicit constraints), and CB (constraint elimination via parameterization), spanning a graduated accuracy–efficiency spectrum, and validate them across five region shapes and two complex-constraint scenarios. (3) We demonstrate that the analytical gradient recovers the full gradient from a single evaluation of the response matrix (versus 2*N* + 1 evaluations for a finite-difference gradient), with a wall-clock speedup growing from 6.3× at *N* = 6 to 30.2× at *N* = 100, and that this reduction in κ(G) translates directly into improved moment tensor inversion accuracy. Together, these contributions convert surface sensor placement from a zero-order sampling problem into a gradient-based, systematically optimizable one, and should be read as a methodology-and-benchmark contribution rather than a field-data study.

The remainder of this paper is organized as follows. Section 2 formulates the optimization problem and presents the analytical gradient derivation along with the three optimization methods. Section 3 provides systematic numerical validation covering convergence behavior, statistical robustness, complex constraints, gradient verification, and synthetic inversion. Section 4 discusses the limitations of this work, and Section 5 concludes the paper.

## 2. Methodology

### 2.1. Forward Model and Problem Formulation

The core of microseismic moment tensor inversion is to infer the mechanical mechanism of a seismic source from P-wave amplitudes recorded by a limited number of sensors. This inference process relies on a forward model, which is entirely determined by the geometric relationship between the source and the sensors. Hence, the spatial configuration of the sensor array directly affects inversion accuracy and reliability through the structure of the forward model.

Consider a point-source seismic event whose mechanical process is described by the seismic moment tensor, a second-order symmetric tensor containing six independent components denoted $M=(M_{11},M_{12},M_{13},M_{22},M_{23},M_{33}{)}^{T}$ [31]. Let the source be at $s=(x_{s},y_{s},z_{s})$ and N sensors be deployed at positions $r_{i}=(x_{i},y_{i},z_{i})$, i=1,…,N. For surface monitoring, the z-coordinates of all sensors take the same deployment elevation $z_{\mathrm{surface}}$. However, the theoretical framework can be extended to downhole, deep sensor arrays, or hybrid deployment networks at any known elevation.

Under the far-field approximation of a homogeneous isotropic medium, the radial component of the P-wave displacement at the i-th sensor relates linearly to the moment tensor components [21,24]:

(1) $$u=G\left(r_{1},\ldots ,r_{N};s\right)\cdot M$$

Here ***G*** ∈ R*^N^*^×6^ denotes the dynamic response (design) matrix, whose i-th row maps the six independent moment tensor components to the P-wave amplitude observed at the i-th sensor; its exact construction is given below in terms of the direction cosines.

Define the relative position vector $\Delta r_{i}=r_{i}-s=(\Delta x_{i},\Delta y_{i},\Delta z_{i})$, distance $R_{i}={∥\Delta r_{i}∥}_{2}$, and direction cosines:

(2) $$r_{1}^{\left(i\right)}=\frac{△x_{i}}{R_{i}},r_{2}^{\left(i\right)}=\frac{△y_{i}}{R_{i}},r_{3}^{\left(i\right)}=\frac{△z_{i}}{R_{i}}$$

The unweighted response row vector for sensor i is:

(3) $$A_{i}=\frac{1}{R_{i}}\left[{\left(r_{1}^{\left(i\right)}\right)}^{2},2r_{1}^{\left(i\right)}r_{2}^{\left(i\right)},2r_{1}^{\left(i\right)}r_{3}^{\left(i\right)},{\left(r_{2}^{\left(i\right)}\right)}^{2},2r_{2}^{\left(i\right)}r_{3}^{\left(i\right)},{\left(r_{3}^{\left(i\right)}\right)}^{2}\right]$$

For vertical component records, multiplying by the radiation pattern factor $\gamma_{3}^{\left(i\right)}$ yields the weighted response $G_{i}=\gamma_{3}^{\left(i\right)}\cdot A_{i}$. Stacking the N row vectors forms $G=[G_{1}^{T},\ldots ,G_{N}^{T}{]}^{T}\in R^{N\times 6}$.

In actual observations, P-wave amplitude picking is inevitably contaminated by background noise and instrumental noise. Let the observed amplitude at the i-th sensor be $u_{i}^{\mathrm{obs}}=u_{i}+\epsilon_{i}$, where $\epsilon_{i}$ denotes the amplitude picking error. Writing the observation equations in vector form: $u^{\mathrm{obs}}=GM+\epsilon$, where ϵ is the noise vector. Under the assumption of mutually independent amplitude-picking errors with equal variance, $\mathrm{Cov}(\epsilon )=\sigma^{2}I_{N}$. The least-squares estimate is $\hat{M}=G^{†}u^{\mathrm{obs}}=(G^{T}G{)}^{-1}G^{T}u^{\mathrm{obs}}$, where $G^{†}$ denotes the Moore–Penrose pseudoinverse, assuming N≥6 and G has full column rank. Substituting the forward model yields $\hat{M}=M+G^{†}\epsilon$, then the estimation error is entirely determined by the amplification of noise through $G^{†}$. The noise amplification is quantified by the condition number:

(4) $$\kappa \left(G\right)=\frac{\sigma_{\max}\left(G\right)}{\sigma_{\min}\left(G\right)}$$

The classical error bound [32]:

(5) $$\frac{{\left\|\hat{M}-M\right\|}_{2}}{{\left\|M\right\|}_{2}}\leq \kappa \left(G\right)\cdot \frac{{\left\|\varepsilon \right\|}_{2}}{{\left\|GM\right\|}_{2}}$$

establishes the theoretical basis for minimizing κ(G). This upper bound is tight under the worst-case noise distribution [32], and there exists a noise vector ϵ such that equality holds. From a Bayesian experimental design perspective, the posterior covariance matrix of the least-squares estimate is $\sigma^{2}(G^{T}G{)}^{-1}$, whose spectral norm condition number is proportional to $\kappa (G{)}^{2}$, minimizing κ(G) and, therefore, also minimizing the worst-case upper bound of posterior uncertainty [17].

The noise model above assumes Cov(ε) = σ^2^I_N_, i.e., independent, identically distributed Gaussian errors with a spherical covariance. The framework generalizes to more realistic noise structures. For a diagonal but non-spherical covariance Cov(ε) = diag(σ_1_^2^, …, σ_N_^2^), the appropriate estimator is the weighted least-squares solution, and the design objective becomes the condition number of the weighted matrix WG with W = diag(1/σ_i_). The analytical gradient derivation carries over unchanged because κ(WG) is again a smooth function of the sensor positions through the same direction-cosine chain rule. For correlated noise, Cov(ε) = C, pre-whitening with C^−1/2^ reduces the problem to the spherical case, so the objective becomes κ(C^−1/2^G), and the gradient follows identically. Non-Gaussian noise (e.g., heavy-tailed amplitude-picking errors) falls outside the least-squares framework; robust estimators would replace the least-squares objective, which we leave to future work.

The optimization problem is formulated as:

(6) $$\begin{array}{l} \underset{r_{1},\ldots ,r_{N}}{\min}\kappa \left(G\left(r_{1},\ldots r_{N};s\right)\right)\\ s.t.\;r_{i}\in \Omega ,i=1,\ldots N \end{array}$$

The mathematical properties of κ(G) have decisive influence on algorithm choice. First, κ(G) is non-convex, with each element of G depending nonlinearly on sensor coordinates through direction cosines—the fundamental reason why coordinate descent methods easily become trapped in inferior local optima. Second, in regions where G has full column rank ($\sigma_{min}>0$), κ is continuously differentiable [32], providing the theoretical basis for gradient-based methods. Third, the problem exhibits rotational symmetry: a global rotation preserves relative geometry, leading to non-unique optimal solutions—any global rotation of an optimal configuration remains optimal. Fourth, when two sensor positions coincide, G acquires approximately duplicate rows, $\sigma_{min}\to 0$ and κ→∞, forming natural “penalty barriers” that help repel the optimization trajectory from ill-conditioned configurations. For surface monitoring, $z_{i}=z_{\mathrm{surface}}$ reduces continuous variables to 2N.

### 2.2. Analytical Gradient of the Condition Number

Let $G=U\Sigma V^{T}$ be the SVD with singular values $\sigma_{1}\geq \ldots \geq \sigma_{6}>0$, where $U=\left[u_{1},\ldots ,u_{N}\right]\in {\mathbb{R}}^{N\times N}$, $V=\left[v_{1},\ldots ,v_{6}\right]\in {\mathbb{R}}^{6\times 6}$, and $\Sigma =\mathrm{diag}\left(\sigma_{1},\ldots ,\sigma_{6}\right)$. For a small perturbation δp of parameter p (any sensor coordinate $x_{i}$ or $y_{i}$), the first-order variation in the k-th singular value is given by singular value perturbation theory [32,33]. The derivation follows from taking the total differential of $\sigma_{k}=u_{k}^{T}Gv_{k}$: $\partial \sigma_{k}/\partial p=u_{k}^{T}\left(\partial G/\partial p\right)v_{k}+\left(\partial u_{k}^{T}/\partial p\right)Gv_{k}+u_{k}^{T}G\left(\partial v_{k}/\partial p\right)$. Using $Gv_{k}=\sigma_{k}u_{k}$ and $u_{k}^{T}G=\sigma_{k}v_{k}^{T}$, the latter two terms reduce to $\sigma_{k}\left(\partial u_{k}^{T}/\partial p\right)u_{k}$ and $\sigma_{k}v_{k}^{T}\left(\partial v_{k}/\partial p\right)$, both of which vanish identically from the unit-norm conditions $u_{k}^{T}u_{k}=1$ and $v_{k}^{T}v_{k}=1$. This yields the remarkably simple result:

(7) $$\frac{\partial \sigma_{k}}{\partial p}=u_{k}^{T}\frac{\partial G}{\partial p}v_{k}$$

Geometrically, Equation (7) states that the derivative of any singular value equals the projection of ∂G/∂p onto the subspace spanned by the corresponding left and right singular vectors, revealing the intrinsic connection between gradient information and SVD.

Applying the quotient rule to $\kappa =\sigma_{1}/\sigma_{6}$:

(8) $$\frac{\partial \kappa }{\partial p}=\frac{1}{\sigma_{6}}\frac{\partial \sigma_{1}}{\partial p}-\frac{\sigma_{1}}{\sigma_{6}^{2}}\frac{\partial \sigma_{6}}{\partial p}$$

The sign structure of Equation (8) has a clear physical interpretation: reducing κ requires the simultaneous shrinking of the largest singular value ($\partial \sigma_{1}/\partial p<0$) and enlarging of the smallest value ($\partial \sigma_{6}/\partial p>0$), with the two contributions weighted by $1/\sigma_{6}$ and $\sigma_{1}/\sigma_{6}^{2}$, respectively. When the matrix is nearly singular ($\sigma_{6}\ll \sigma_{1}$), the weight is $\sigma_{1}/\sigma_{6}^{2}\gg 1/\sigma_{6}$, implying that enlarging the smallest singular value—i.e., improving the weakest direction of the observation system—is the dominant pathway to reducing the condition number. Substituting Equation (7) yields the gradient formula:

(9) $$\nabla_{\left(x,y\right)}\;\kappa =\frac{1}{\sigma_{6}}u_{1}^{T}\left(\nabla G\right)v_{1}-\frac{\kappa }{\sigma_{6}}u_{6}^{T}\left(\nabla G\right)v_{6}$$

Equation (9) reduces the gradient computation to the matrix derivatives ∂G/∂p. Since each row of $G_{i}$ depends only on sensor i, the derivative $\partial G/\partial x_{i}$ is zero except in row i. This sparsity allows all 2N partial derivatives to be computed independently. The elementary derivatives of direction cosines and distance follow directly from differentiating the definitions $\gamma_{k}^{\left(i\right)}=\Delta_{k}/R_{i}$:

(10) $$\begin{array}{l} \frac{\partial R_{i}}{\partial x_{i}}=\gamma_{1}^{\left(i\right)},\frac{\partial R_{i}}{\partial y_{i}}=\gamma_{2}^{\left(i\right)}\\ \frac{\partial \gamma_{1}^{\left(i\right)}}{\partial x_{i}}=\frac{1-{\left(\gamma_{1}^{\left(i\right)}\right)}^{2}}{R_{i}},\frac{\partial \gamma_{1}^{\left(i\right)}}{\partial y_{i}}=-\frac{\gamma_{1}^{\left(i\right)}\gamma_{2}^{\left(i\right)}}{R_{i}}\\ \frac{\partial \gamma_{2}^{\left(i\right)}}{\partial x_{i}}=-\frac{\gamma_{1}^{\left(i\right)}\gamma_{2}^{\left(i\right)}}{R_{i}},\frac{\partial \gamma_{2}^{\left(i\right)}}{\partial y_{i}}=\frac{1-{\left(\gamma_{2}^{\left(i\right)}\right)}^{2}}{R_{i}}\\ \frac{\partial \gamma_{3}^{\left(i\right)}}{\partial x_{i}}=-\frac{\gamma_{1}^{\left(i\right)}\gamma_{3}^{\left(i\right)}}{R_{i}},\frac{\partial \gamma_{3}^{\left(i\right)}}{\partial y_{i}}=-\frac{\gamma_{2}^{\left(i\right)}\gamma_{3}^{\left(i\right)}}{R_{i}} \end{array}$$

Each element of the unweighted row vector $A_{i}$ has the form $A_{ij}=N_{ij}\left(\gamma_{1}^{\left(i\right)},\gamma_{2}^{\left(i\right)},\gamma_{3}^{\left(i\right)}\right)/R_{i}$, where $N_{ij}$ is a homogeneous polynomial of the direction cosines:

(11) $$N_{i1}={\left(\gamma_{1}^{\left(i\right)}\right)}^{2},N_{i2}=2\gamma_{1}^{\left(i\right)}\gamma_{2}^{\left(i\right)},N_{i3}=2\gamma_{1}^{\left(i\right)}\gamma_{3}^{\left(i\right)}$$

(12) $$N_{i4}={\left(\gamma_{2}^{\left(i\right)}\right)}^{2},N_{i5}=2\gamma_{2}^{\left(i\right)}\gamma_{3}^{\left(i\right)},N_{i6}={\left(\gamma_{3}^{\left(i\right)}\right)}^{2}$$

Differentiating with respect to the coordinate $p\in \left\{x_{i},y_{i}\right\}$ via the quotient rule:

(13) $$\frac{\partial A_{ij}}{\partial p}=\frac{1}{R_{i}}\frac{\partial N_{ij}}{\partial p}-\frac{A_{ij}}{R_{i}}\frac{\partial R_{i}}{\partial p}$$

where $\partial N_{ij}/\partial p$ uses the chain rule with Equation (13). For the P-wave vertical component, the weighted row vector is $G_{i}=\gamma_{3}^{\left(i\right)}A_{i}$, and by the product rule:

(14) $$\frac{\partial G_{i}}{\partial p}=\frac{\partial \gamma_{3}^{\left(i\right)}}{\partial p}A_{i}+\gamma_{3}^{\left(i\right)}\frac{\partial A_{i}}{\partial p}$$

Computational cost: The dominant cost is the SVD of G (N×6), requiring $O\left(36N\right)$ operations [32]. Computing ∂G/∂p for all 2N coordinates via Equations (12)–(14) costs $O\left(N\right)$, and the projections in Equation (10) cost $O\left(12N^{2}\right)$. For N=6, the gradient computation overhead relative to the SVD is less than 5%, meaning the full gradient vector is obtained at negligible additional cost beyond the SVD already required for condition number evaluation.

The derivation assumes that **G** has full column rank with simple singular values. Two edge cases lie outside this assumption, and neither affects the reported results. Numerical instability as σ_min_ approaches zero cannot occur here: moment tensor inversion requires **G** to have full column rank, so σ_min_ is strictly positive and κ = σ_max_/σ_min_ is at least 1, and the gradient stays finite in every configuration the method can operate on. A repeated singular value, where the gradient is undefined, requires exact geometric symmetry, for instance, six sensors on the vertices of a perfect regular polygon with the source directly below its center. Exact coincidence of this kind is a measure-zero event that finite-precision optimization never reaches. Near the coincidence, the two singular values differ, and the gradient is defined, with only a slight numerical instability in the orientation of the singular vectors.

### 2.3. BFGS Quasi-Newton with Quadratic Penalty (BP)

BP converts the constrained problem via a quadratic penalty function:

(15) $$\Phi_{\mu }\left(x\right)=\kappa \left(G\left(x\right)\right)+\mu \overset{N}{\underset{i=1}{\sum }}{\left[\max\left(0,c_{i}\left(x\right)\right)\right]}^{2}$$

where $x\in {\mathbb{R}}^{2N}$ concatenates all sensor coordinates, $c_{i}\left(x\right)\leq 0$ is the constraint function, and μ>0 is the penalty parameter. For a circle of radius $R_{\max}$, $c_{i}=\sqrt{{\left(x_{i}-x_{c}\right)}^{2}+{\left(y_{i}-y_{c}\right)}^{2}}-R_{\max}$.

The BFGS method [33] maintains an inverse Hessian approximation $H_{k}\in {\mathbb{R}}^{2N\times 2N}$, updated via:

(16) $$\begin{array}{c} p_{k}=-H_{k}\nabla \Phi_{\mu }\left(x_{k}\right)\\ \alpha_{k}=\mathrm{Arm}\left(c_{1}\right),c_{1}=10^{-4}\;\\ x_{k+1}=x_{k}+\alpha_{k}p_{k}\\ s_{k}=x_{k+1}-x_{k},y_{k}=\nabla \Phi_{\mu }\left(x_{k+1}\right)-\nabla \Phi_{\mu }\left(x_{k}\right)\\ \rho_{k}=1/\left(y_{k}^{T}s_{k}\right)\\ H_{k+1}=\left(I-\rho_{k}s_{k}y_{k}^{T}\right)H_{k}\left(I-\rho_{k}y_{k}s_{k}^{T}\right)+\rho_{k}s_{k}s_{k}^{T} \end{array}$$

where $\mathrm{Arm}\left(c_{1}\right)$ denotes the Armijo line search rule that selects a step size via the sufficient decrease criterion with parameter *c*_1_.

**Figure 1:**
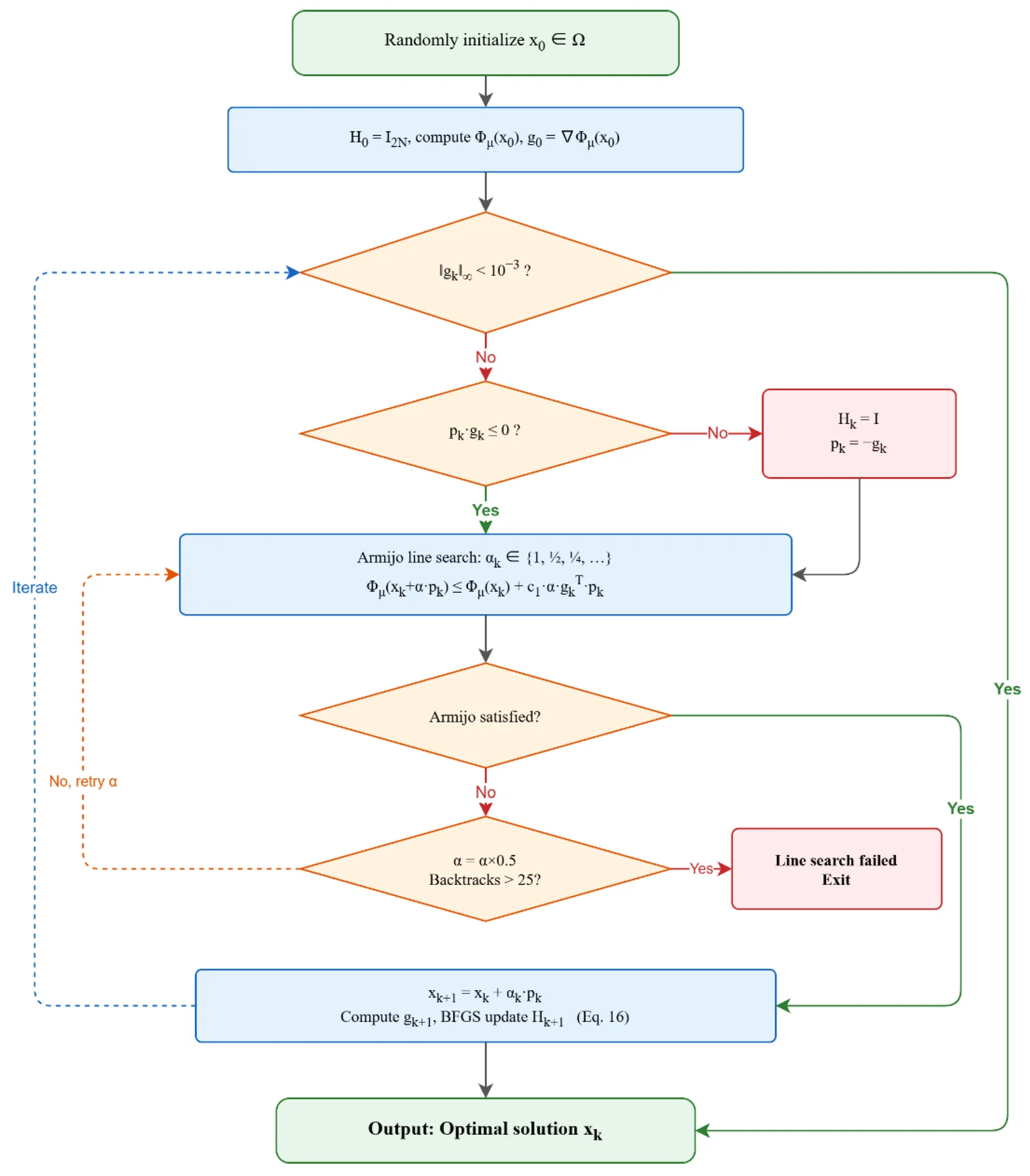
BP algorithm flowchart.

BP’s penalty parameter is set to μ=50 as a compromise between constraint satisfaction and numerical conditioning. A brief sensitivity analysis on a circular region illustrates the trade-off. With μ=10, constraint violations of up to 15% of $R_{\max}$ occur in early iterations before being corrected, yielding κ=46.1 (6% above the optimum); with μ=100, violations are reduced to <1% but the Hessian condition number increases by a factor of ~3, slowing BFGS convergence by ~30%; and with μ=500, the problem becomes numerically ill-conditioned, and the line search frequently fails. The value μ=50 balances constraint satisfaction against numerical conditioning for the geometries tested. The soft-boundary approach allows sensors to temporarily cross the boundary during iterations, which facilitates exploration of the search space, which is an advantage over hard-boundary methods in early iterations when the optimizer is far from the feasible region. The BP algorithm flowchart is shown in Figure 1.

However, BP’s fundamental limitation is the differentiability requirement of the constraint function. For a circular region, the constraint $c_{i}=\sqrt{{\left(x_{i}-x_{c}\right)}^{2}+{\left(y_{i}-y_{c}\right)}^{2}}-R_{\max}$ is $C^{\infty }$ everywhere, and the penalty gradient $\partial \Phi_{\mu }/\partial x_{i}$ is well-defined. But for a polygon with L sides, at any vertex the directional derivative of $c_{i}\left(\theta \right)$ is discontinuous, jumping from the outward normal of one edge to that of another. When a sensor approaches a vertex, the penalty gradient direction is distorted by up to the exterior angle of the polygon (e.g., 60° for a triangle), and the Armijo line search fails to satisfy the sufficient decrease condition even after the maximum backtracking steps. Consequently, BP works reliably only on $C^{1}$-smooth regions such as circles and squares, but prematurely terminates on general polygonal domains. This theoretical prediction is directly confirmed by the numerical experiments in Section 3.

### 2.4. Sequential Quadratic Programming (FMC/SQP)

SQP retains constraints as explicit nonlinear inequalities and solves a quadratic programming subproblem at each iteration:

(17) $$\begin{array}{c} \underset{d}{\min}\frac{1}{2}d^{T}H_{k}d+\nabla \kappa {\left(x_{k}\right)}^{T}d\\ s.t.\;c_{i}\left(x_{k}\right)+\nabla c_{i}{\left(x_{k}\right)}^{T}\;d\leq 0,i=1,\ldots ,N \end{array}$$

The QP subproblem uses an active-set method (feasibility tolerance $10^{-8}$). The merit function is the $l_{1}$ exact penalty:

(18) $$\phi_{1}\left(x;\nu \right)=\kappa \left(x\right)+\nu \overset{N}{\underset{i=1}{\sum }}\max\left(0,c_{i}\left(x\right)\right)$$

with penalty parameter $\nu_{k}$ updated at each iteration to satisfy $\nu_{k}>{∥\lambda_{k}∥}_{\infty }$, where $\lambda_{k}$ are the Lagrange multipliers from the QP solution. This condition ensures that $d_{k}$ is a descent direction for $\phi_{1}$. The line search uses the Armijo backtracking condition applied to $\phi_{1}$ with $c_{1}=10^{-4}$ and backtracking factor 0.5, truncated at 15 steps. The BFGS update uses the damped formula on the Lagrangian Hessian to maintain positive definiteness when $y_{k}^{T}s_{k}\leq 0$ would otherwise violate the curvature condition. Convergence is declared when ${∥d_{k}∥}_{\infty }<10^{-6}$ and $\max_{i}c_{i}\left(x_{k}\right)<10^{-8}$.

**Figure 2:**
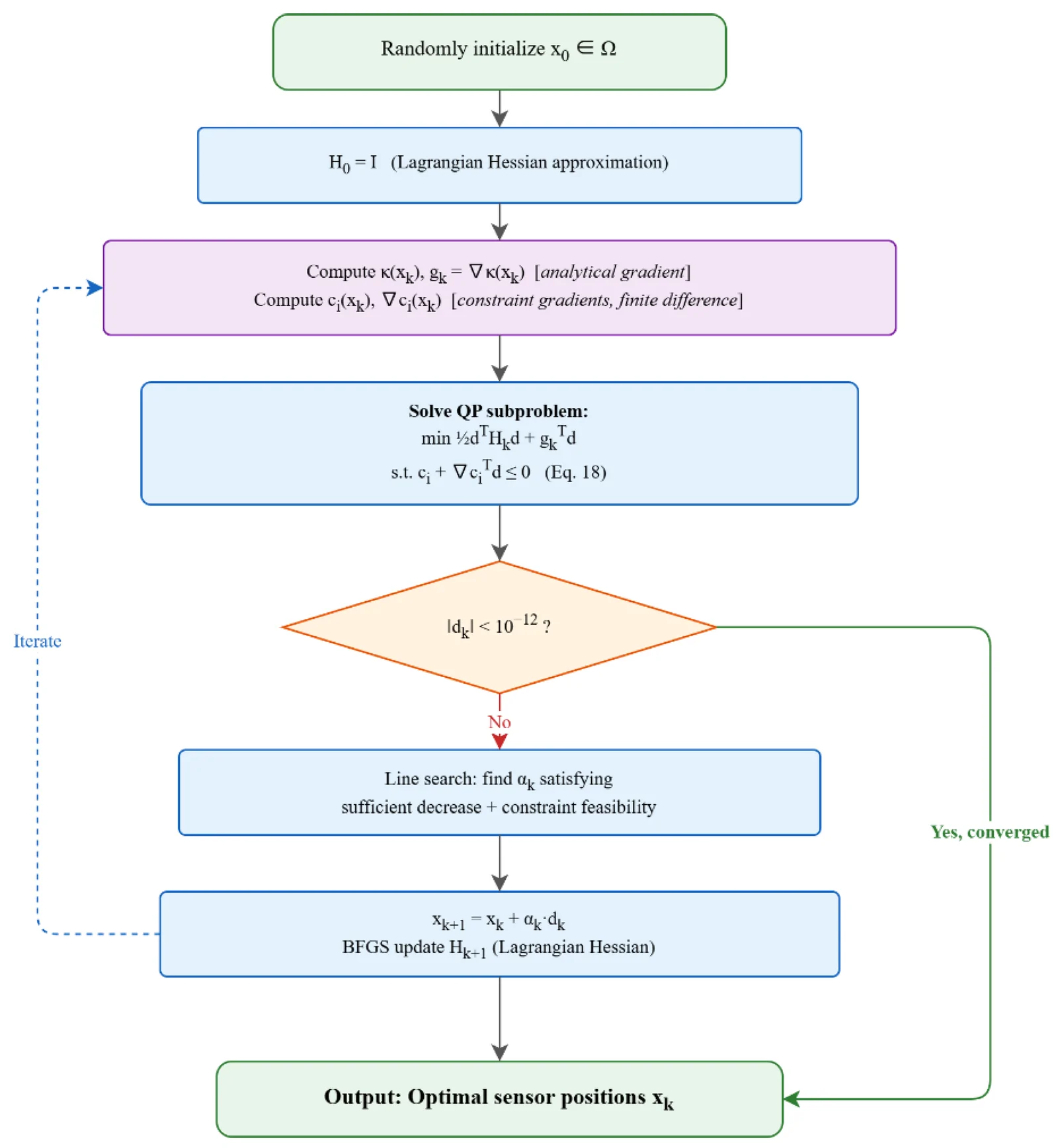
FMC algorithm flowchart.

FMC’s hard-boundary approach guarantees constraint satisfaction within the specified tolerance. The QP subproblem simultaneously accounts for objective curvature and constraint geometry, and at each iteration, the quadratic approximation $\frac{1}{2}d^{T}H_{k}d+\nabla \kappa {\left(x_{k}\right)}^{T}d$ captures the local curvature of the objective, while the linearized constraints $c_{i}\left(x_{k}\right)+\nabla c_{i}{\left(x_{k}\right)}^{T}d\leq 0$ ensure first-order feasibility. This enables superlinear convergence near the constrained optimum as the BFGS Hessian accumulates sufficient curvature information. FMC works reliably on all shapes because its constraint gradients are computed via finite differences—independent of whether the constraint functions themselves are analytically differentiable—and the SQP framework enjoys theoretical global convergence guarantees under standard assumptions [33]. The cost is the highest among gradient methods: approximately 5000 evaluations per optimization due to constraint gradient computation (2N function perturbations per constraint) and QP subproblem solving ($O\left(N^{2}\right)$ per iteration). This cost, however, yields the most accurate and reliable results across all region geometries, as demonstrated in Section 3. The FMC algorithm flowchart is shown in Figure 2.

### 2.5. Center-Boundary Parameterization (CB)

Kong et al. [24] observed a striking regularity in systematic numerical experiments: regardless of the initial configuration and regardless of whether the region shape is circular or polygonal, optimal sensor configurations consistently place sensors at two types of extreme locations—the region center (typically 1 sensor) and the region boundary (the remaining sensors). This “center-boundary” structure is not a coincidental numerical artifact but has a clear geometric root. Condition number minimization requires maximal linear independence among the row vectors of G, and the differences among row vectors arise from the differences in direction cosines $\left(\gamma_{1}^{\left(i\right)},\gamma_{2}^{\left(i\right)},\gamma_{3}^{\left(i\right)}\right)$ of the sensors. Boundary sensors maximize the horizontal angular deviation from the source-sensor line relative to the vertical direction, producing the widest possible spread of $\left(\gamma_{1}^{\left(i\right)},\gamma_{2}^{\left(i\right)}\right)$ values; the center sensor provides a reference vector nearly orthogonal to the direction cosines of boundary sensors ($\gamma_{1},\gamma_{2}\approx 0$, $\gamma_{3}\approx 1$). Together, these effects spread the row vectors of G as widely as possible in ${\mathbb{R}}^{6}$, precisely the geometric configuration that minimizes the condition number. A quantitative illustration makes this mechanism concrete. A sensor at the region center has horizontal direction cosines (γ_1_, γ_2_) ≈ (0, 0) and a near-vertical reference vector (γ_3_ ≈ 1). A sensor at distance d from the center along the boundary instead has $\sqrt{\left(\gamma_{1}^{2}+\gamma_{2}^{2}\right)}\approx d/R$, so its row direction deviates maximally from the center sensor’s, and the two rows are close to orthogonal. Moving a sensor from the interior toward the boundary therefore monotonically increases the angular spread of the direction-cosine vectors and hence the linear independence of the rows of G. This is the same mechanism by which the condition number decreases. This argument explains, without constituting a formal proof, why boundary-concentrated configurations are consistently favored. A key practical corollary follows: optimal sensors are empirically observed, across all tested shapes and initial conditions, to concentrate on (or very close to) the boundary. We give a geometric rationale for this observation below, but do not claim a rigorous proof. The optimizer can therefore, for the configurations studied here, restrict its search to the boundary.

With $n_{c}$ center sensors and $n_{b}=N-n_{c}$ boundary sensors, each boundary sensor is parameterized by a single angle $\theta_{i}$:

(19) $$x_{i}\left(\theta_{i}\right)=x_{c}+R_{\mathrm{boundary}}\left(\theta_{i}\right)\cos\theta_{i},\;y_{i}\left(\theta_{i}\right)=y_{c}+R_{\mathrm{boundary}}\left(\theta_{i}\right)\sin\theta_{i}$$

For a circle, $R_{\mathrm{boundary}}\left(\theta \right)\equiv R_{\max}$. For an L-sided convex polygon with angular spacing Δα=2π/L:

(20) $$R_{\mathrm{boundary}}\left(\theta \right)=\frac{R_{\max}}{\cos\varphi +\sin\varphi /\tan\left(\left(\pi -\Delta \alpha \right)/2\right)}$$

where φ is the angular distance from θ to the perpendicular bisector of the nearest edge. Variables reduce from 2N Cartesian coordinates to $n_{b}$ angular parameters (for N=6 with 1 center sensor from 12 to 5). The chain-rule gradient is as follows:

(21) $$\frac{\partial \kappa }{\partial \theta_{i}}=\frac{\partial \kappa }{\partial x_{i}}\frac{\partial x_{i}}{\partial \theta_{i}}+\frac{\partial \kappa }{\partial y_{i}}\frac{\partial y_{i}}{\partial \theta_{i}}$$

with $\partial x_{i}/\partial \theta_{i}=-R_{\mathrm{boundary}}\left(\theta_{i}\right)\sin\theta_{i}$ and $\partial y_{i}/\partial \theta_{i}=R_{\mathrm{boundary}}\left(\theta_{i}\right)\cos\theta_{i}$. The number of center sensors must satisfy $N-n_{c}\leq L$ to prevent boundary sensor redundancy (for the triangle, L=3 requires $n_{c}=3$). Standard BFGS without penalty terms is applied to the angular variables.

**Figure 3:**
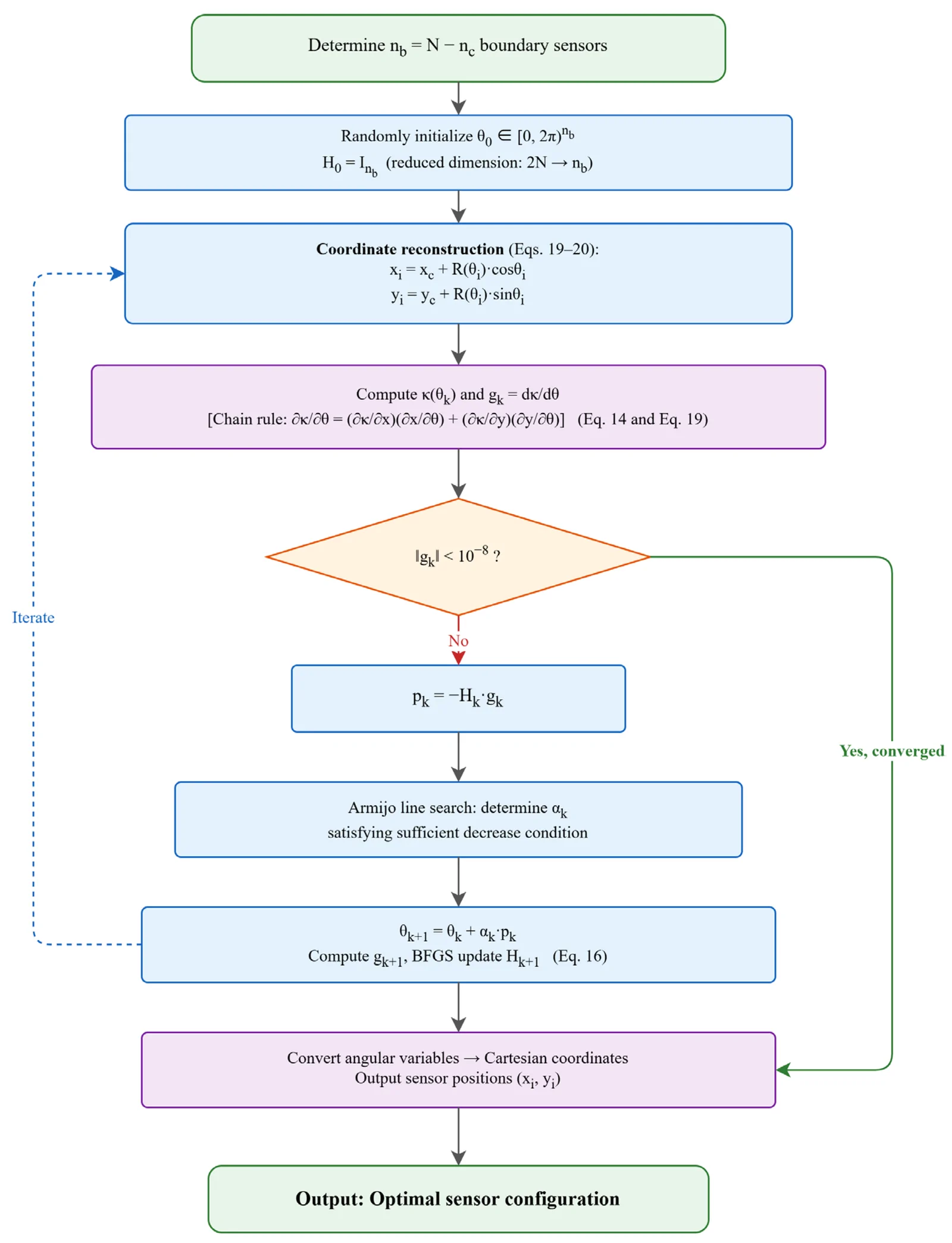
CB algorithm flowchart.

CB’s constraint strategy is fundamentally different from BP and FMC: rather than handling constraints at the algorithmic level, it eliminates them through variable substitution. Regardless of $\theta_{i}$, the reconstructed Cartesian coordinates $x_{i}=x_{c}+R_{\mathrm{boundary}}\left(\theta_{i}\right)\cos\theta_{i}$ and $y_{i}=y_{c}+R_{\mathrm{boundary}}\left(\theta_{i}\right)\sin\theta_{i}$ always lie exactly on the boundary—the constraint mathematically ceases to exist. The benefits are threefold: dimensionality reduction (12→5 for N=6 with 1 center sensor), which shrinks the search space volume by orders of magnitude; complete elimination of penalty parameters, QP subproblems, and constraint gradient computations; and faster BFGS convergence due to improved Hessian conditioning in the lower-dimensional space (5×5 versus 12×12). The cost is structural rigidity: sensors are restricted to the boundary and cannot represent configurations at arbitrary interior positions. When the true optimum places sensors slightly inside the boundary (as FMC occasionally finds on certain shapes), CB’s κ is 10–20% higher—an accuracy penalty that must be weighed against its 16× evaluation-count advantage. The CB algorithm flowchart is shown in Figure 3.

The choice of the number of center sensors $n_{c}$ depends on N and the region shape. For an L-sided polygon, to avoid boundary sensor redundancy (multiple sensors sharing the same edge normal direction, which would cause near-linear dependence among rows of G), one must ensure $N-n_{c}\leq L$, i.e., $n_{c}\geq \max\left(0,N-L\right)$. For N=6 and a triangle (L=3), this requires $n_{c}=3$, leaving only 3 boundary sensors distributed over 3 edges—a constraint that, if violated, leads to $\mathrm{rank}\left(G\right)<6$ and κ→∞, as confirmed experimentally in Section 3.2.

The CB framework extends naturally to non-simply connected regions. For an annular region (outer radius $R_{\mathrm{out}}$, inner radius $R_{\mathrm{in}}$), all N sensors are allocated to the two boundaries since the center is inaccessible. Each sensor is assigned to either the outer or inner boundary, with the inner-outer allocation ratio as an additional discrete decision variable: $x_{i}=x_{c}+R_{\mathrm{out}}\cos\theta_{i}$ or $x_{i}=x_{c}+R_{\mathrm{in}}\cos\theta_{i}$. BFGS optimizes the angular positions, and the allocation ratio is determined by exhaustive enumeration over the N+1 possible splits. For disconnected patches (P circular regions of potentially different radii and centers), the problem acquires a discrete combinatorial dimension: an exhaustive search over all integer partitions of N sensors into P patches (for N=6, P=2: 7 partitions) resolves the discrete allocation, with BFGS independently optimizing angular positions within each patch. For the small instances considered here (N = 6 sensors, P = 2 patches, seven integer partitions), the exhaustive enumeration guarantees global optimality of the discrete allocation at moderate additional cost. The exhaustive strategy scales combinatorially with the number of sensors and patches and is not intended for large N or many patches. For such cases, heuristic partition search, branch-and-bound, or two-level optimization are natural extensions (see Section 4).

## 3. Numerical Validation

### 3.1. Experimental Setup

All experiments use N=6 surface sensors at elevation z=1000 m, monitoring a source located at $s=\left(1000,0,0\right)$ m. The P-wave vertical component is used. The region scale parameter for all shapes is $R_{\max}=500$ m. Five typical region shapes are tested (circle, square, triangle, hexagon, octagon) plus two complex constraint scenarios (annular region: $R_{\mathrm{in}}=200$ m, $R_{\mathrm{out}}=500$ m; disconnected patches: two circular regions of radius 250 m centered at $\left(\pm 300,0\right)$). OLD adopts the settings from [24] (initial step sizes $\Delta R=R_{\max}/10$ and Δθ=2π/10). BP uses a fixed penalty parameter μ=50. FMC employs the SQP algorithm with analytical objective gradient and finite-difference constraint gradients. CB uses a parameterization of one center sensor plus five boundary sensors (five angular variables) for circles, squares, hexagons, and octagons, and three center sensors for triangles (to satisfy $n_{c}\geq N-L=3$). FDG replaces the analytical gradient with central differences ($h=10^{-6}$) in the BP framework, requiring 13 G evaluations per gradient computation. The evaluation metrics are the final condition number κ, the number of G matrix evaluations $N_{\mathrm{eval}}$, and the standard deviation $\sigma_{\kappa }$ across trials.

The present experiments are subject to four simplifying assumptions, whose limitations are discussed in Section 4: (1) the sensor count is fixed at *N* = 6, which establishes feasibility and method ranking at a single problem size rather than quantifying scaling to large arrays (the computational scaling is analyzed in Section 3.6); (2) the source location is fixed at a single representative depth, so the reported condition numbers are representative rather than exhaustive over source positions; (3) all sensors share a single deployment elevation (surface monitoring), with downhole and hybrid configurations discussed conceptually but not numerically demonstrated; and (4) the medium is homogeneous and isotropic, so a heterogeneous or anisotropic medium would modify the forward Green’s function while leaving the gradient-derivation framework structurally unchanged. Importantly, none of these assumptions limits the validity or the computational reliability of the method itself: the analytical gradient and the three gradient-based strategies remain well-defined and numerically stable across the tested variations in *N*, source position, and deployment elevation, so these assumptions constrain only the generality of the reported numerical values, not the correctness or the convergence of the proposed algorithms. The summary of the four methods is listed in Table 1.

### 3.2. Convergence Behavior and Single-Trial Comparison

**Figure 4:**
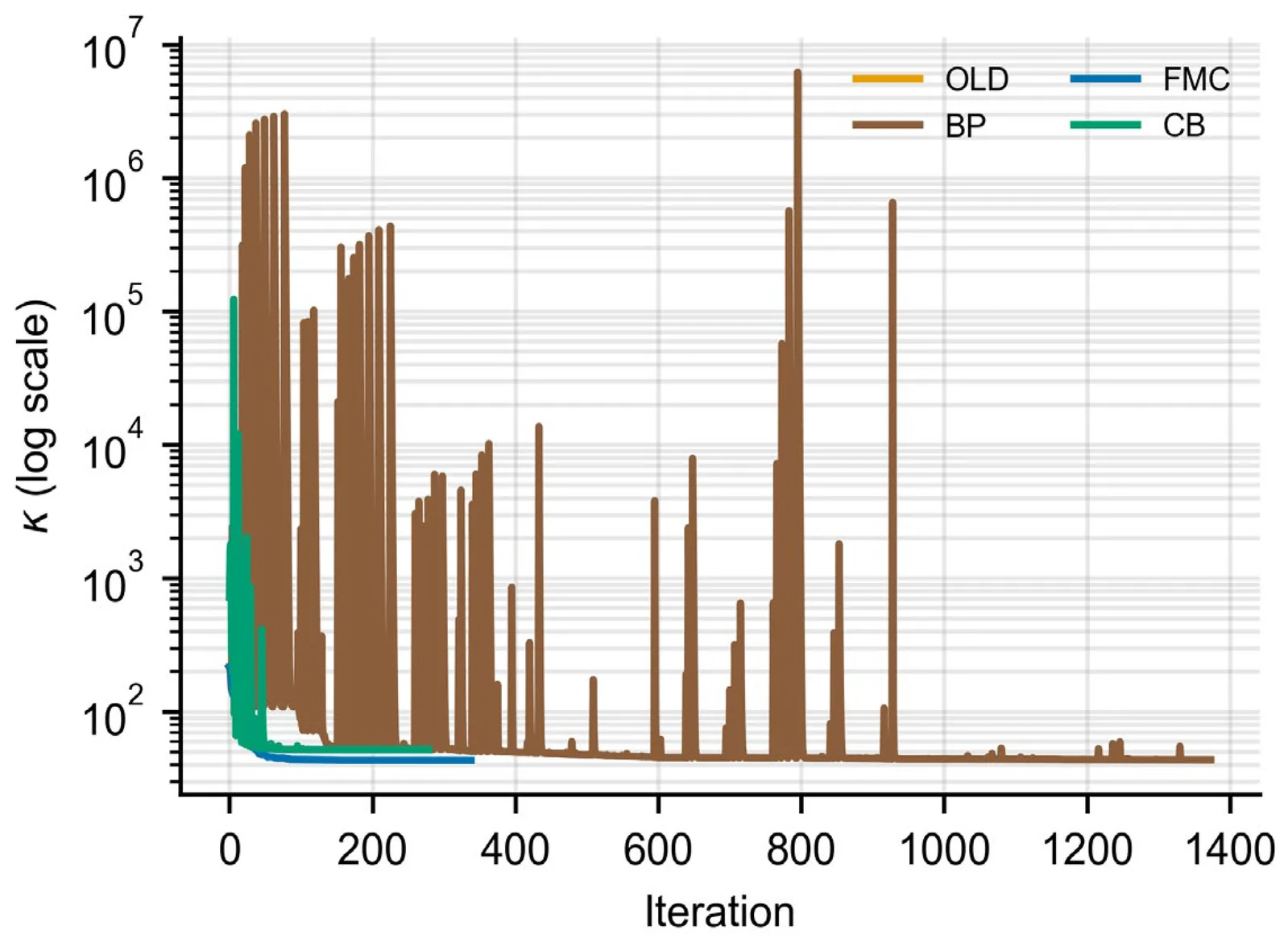
Typical convergence curves of the four methods on a circular region.

To examine intrinsic differences among methods without random-initial-value interference, we compare all four methods starting from the same initial configuration on each shape. Figure 4 shows a typical convergence trajectory on a circular region ($\kappa_{0}=217$). OLD barely moves (217 → 206 over 38 iterations), limited by its four fixed-direction probes being nearly orthogonal to the true gradient. FMC achieves the fastest descent (50–70% reduction in the first 50–100 iterations) and converges to the lowest κ=43.3. BP performs similarly to FMC on this smooth region but fails on polygons when a sensor approaches a non-differentiable vertex. CB converges rapidly due to only five angular variables but plateaus at κ≈52, reflecting its structural assumption that sensors must lie on the boundary.

Table 2 reveals three key findings: (1) OLD is ineffective; the κ reduction never exceeds 3% on any shape, confirming that four fixed-direction probes cannot approximate the true gradient in a 2N-dimensional space. (2) FMC achieves the lowest κ on all shapes (e.g., 37.1 on square, 44.9 on octagon) but requires the most evaluations (~5000). (3) CB offers 5–55× speed advantage on workable shapes but fails on triangles ($\kappa \to 10^{35}$) where $N-n_{c}>L$ causes rank deficiency in G, consistent with the parametric condition derived in Section 2.5. BP performs near FMC on smooth regions (κ=42.9 on circle) but terminates prematurely on polygons (κ=231.6 on triangle), confirming its reliance on constraint differentiability.

**Figure 5:**
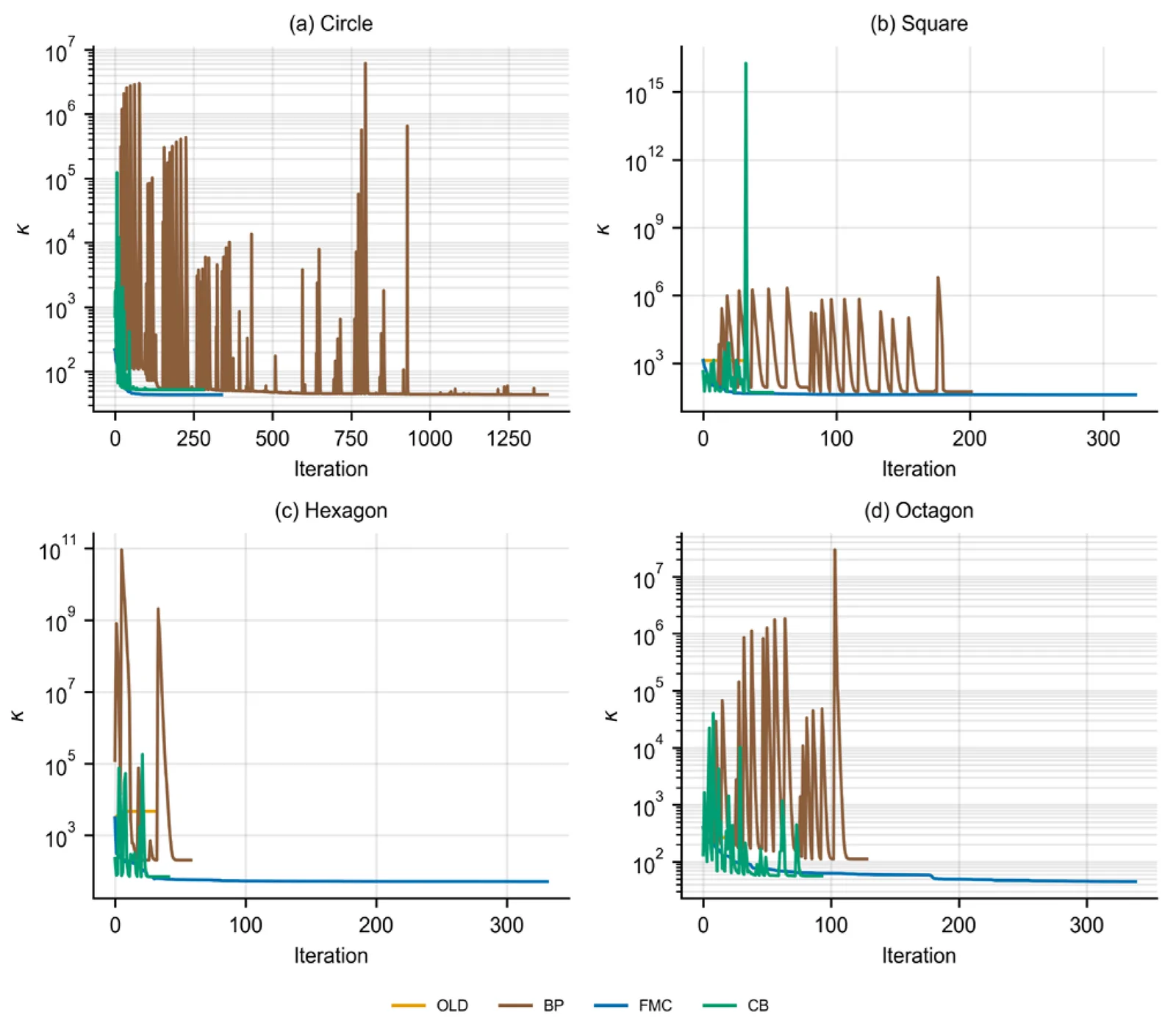
Iterative convergence process on four shapes.

Figure 5 extends Table 2 into full convergence trajectories. OLD remains nearly horizontal across all panels. FMC descends steadily to the lowest κ without premature termination. BP flattens abruptly on hexagon and octagon, marking line-search failure at polygon vertices. CB shows the steepest initial descent but plateaus above FMC’s final value. These trajectories confirm that the failure modes identified in Section 2.3 and Section 2.5—BP at non-differentiable constraints, CB under $N-n_{c}>L$—are systematic rather than random.

### 3.3. Statistical Robustness Across Random Initial Values

To assess statistical reliability across random initial configurations, 30 independent random initial configurations are used per method and per shape (all entries in Table 3 are averaged over these 30 trials). Table 3 reports the mean $\bar{\kappa }$ and standard deviation $\sigma_{\kappa }$. Each entry is the mean and standard deviation over 30 independent random initial configurations per shape.

**Figure 6:**
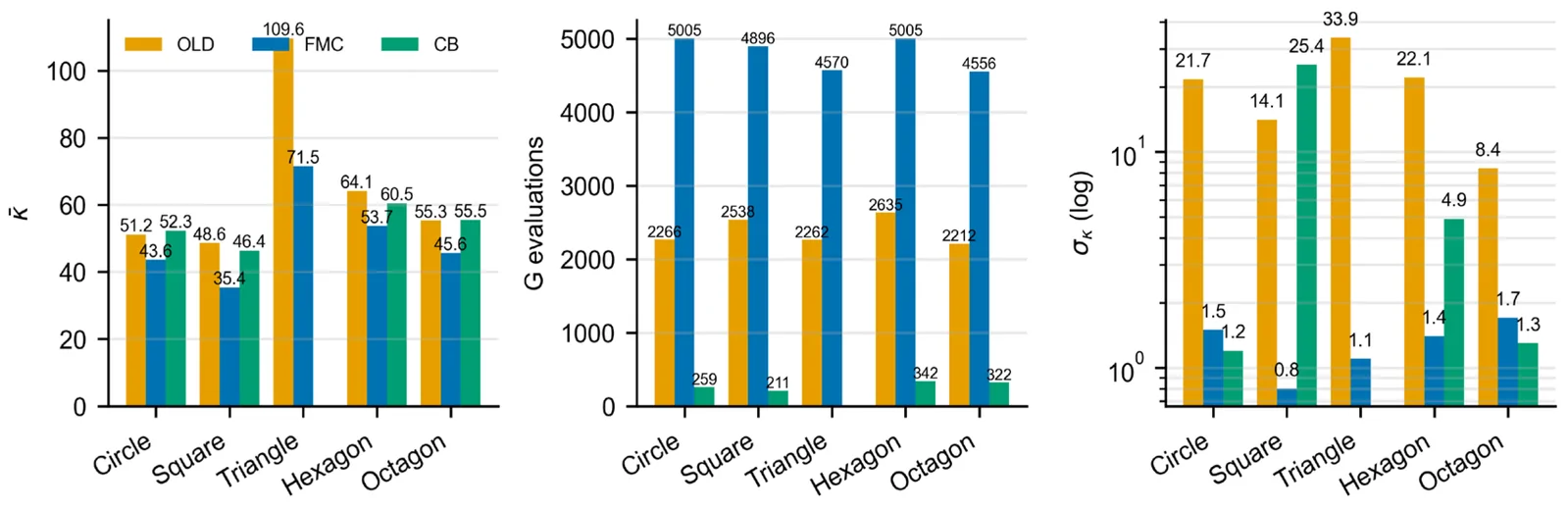
Condition number, evaluation count, and robustness comparison.

FMC achieves the best accuracy ($\bar{\kappa }=49.8$, 25% below OLD) and robustness ($\sigma_{\kappa }=1.3$, a 15.4× reduction from OLD’s 20.0), enabling reliable single-run optimization. CB attains near-optimal quality ($\bar{\kappa }=55.8$) with approximately 295 evaluations (1/16 of FMC), consistent with the 10–20% structural penalty predicted in Section 2.5. BP achieves near-FMC accuracy on smooth regions ($\bar{\kappa }=43.3$ on circle, 36.1 on square) but suffers frequent line-search failures on polygons (marked +). Figure 6 summarizes these three dimensions across all shapes.

It should be noted that missing bars on certain shapes reflect systematic failure modes of the corresponding methods rather than data omission: on the triangle, CB is absent because *N*-*n*_c_ > L causes rank deficiency in **G** ($\bar{\kappa }\to \infty$, see Section 2.5); on the triangle, hexagon, and octagon, BP bars are omitted because the penalty function method fails at polygon vertices where the constraint function is non-differentiable, causing the Armijo line search to fail repeatedly (see Section 2.3). The average rows for BP exclude all three polygons, and CB’s average excludes the triangle.

### 3.4. Re-Analysis of OLD Along Two Dimensions

Because OLD is a zero-order method whose design intent is global exploration, evaluating it under a single-run protocol understates its capability. We therefore report it along two dimensions. For single-run efficiency, one FMC run reaches κ ≈ 43.3 on the circle within about 5000 evaluations, whereas a single OLD run reaches κ ≈ 45.5; FMC is lower on every shape. The data was summarized in Table 4.

For global-search robustness, we give OLD its intended treatment of 100 random starts with best-of-N selection. The results were listed in Table 5.

With its multi-start treatment, OLD’s best-of-100 on the circle (κ ≈ 43.3) is essentially identical to FMC’s single-run result (κ ≈ 43.3), but only at a total cost of about 213,000 evaluations, roughly 43× the budget FMC requires.

### 3.5. Complex Regional Constraints

To assess adaptability beyond simply connected regions, we test an annular region ($R_{\mathrm{in}}=200$ m, $R_{\mathrm{out}}=500$ m) and two disconnected circular patches (R=250 m centered at ±300 m). Table 6 summarizes the results.

**Figure 7:**
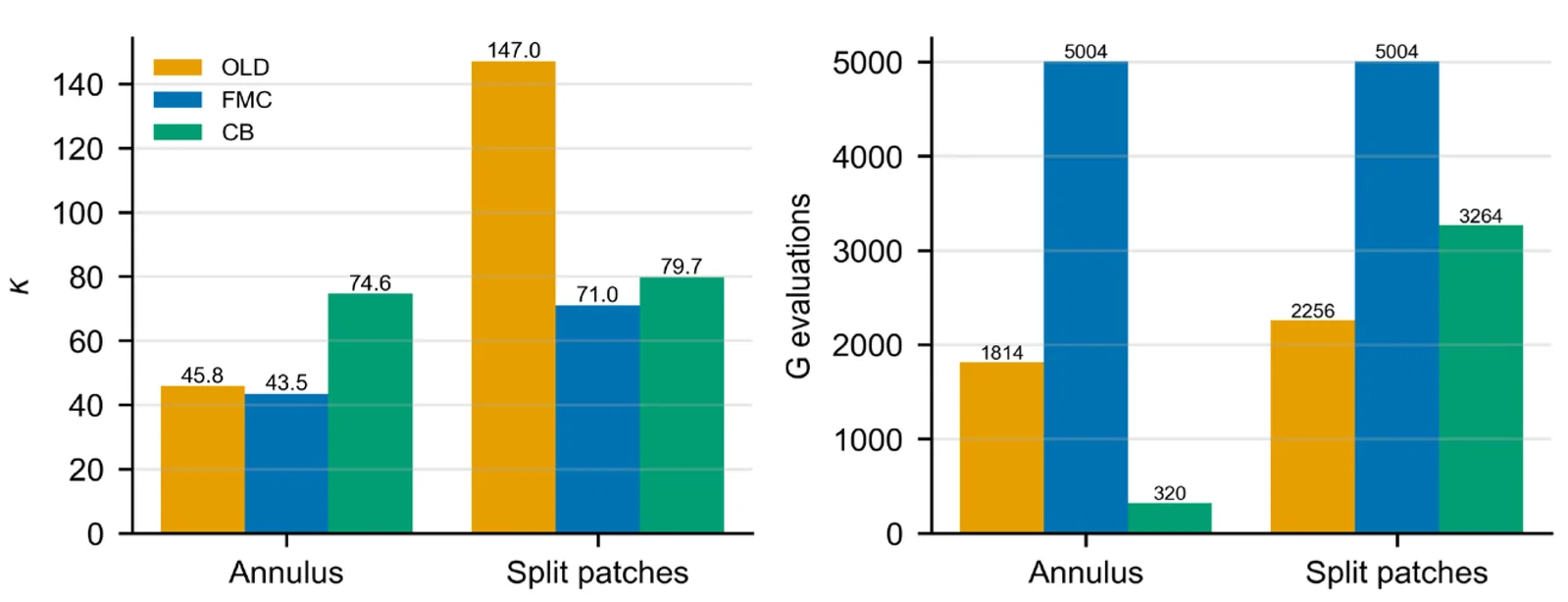
Method comparison on annular and patch regions.

The annular hole has negligible effect (FMC: κ=43.5 vs. 43.6 on the full circle), because optimal configurations do not place sensors near the center—the inner boundary provides comparable direction-cosine diversity. The results are summarized in Figure 7. On disconnected patches, OLD exhibits extreme instability (κ=61–190) since it cannot relocate sensors between patches. FMC converges to κ≈60–70 in 4 of 5 trials, but one trial is trapped at κ=141 due to unfavorable initial patch allocation. CB exhaustively enumerates all seven integer partitions of six sensors into two patches and stably converges to κ=79.7 in every trial. This confirms that discrete sensor-to-subregion allocation must be handled explicitly in disconnected domains and that purely continuous methods cannot cross infeasible gaps.

### 3.6. Computational Scaling

To verify the computational advantage of the analytical gradient at larger problem sizes, we time the analytical gradient against a forward finite-difference gradient at N = 6, 20, 50, 100. The analytical gradient requires one evaluation of G (which also yields κ), while the finite-difference gradient requires 2*N* + 1 evaluations (one base point plus two perturbations per sensor). Median wall-clock times over 20 runs are given in Table 7.

The evaluation count is exactly 1 versus 2N + 1, and the wall-clock speedup grows from 6.3× at N = 6 to 30.2× at N = 100. This confirms the linear-scaling advantage predicted by the cost model. A full single-start fmincon run converges at each size (final κ ≈ 43.8, 38.9, 49.1, and 57.1), so the method remains tractable as N grows.

### 3.7. Gradient Verification and Computational Cost Analysis

To quantify the practical value of the analytical gradient, a finite-difference gradient (FDG) baseline using central differences ($h=10^{-6}$) within the BP framework is benchmarked. Table 8 compares the two approaches.

FDG achieves identical solution quality ($\bar{\kappa }=43.5$ vs. 43.3) but requires 10.4× more G evaluations (15,624 vs. 1496), consistent with the theoretical cost of 2N+1=13 evaluations per gradient via central differences versus <5% overhead for the analytical method. This advantage scales linearly with N, reaching approximately 200× for N=100. Component-wise verification of the analytical gradient against central differences yields a maximum relative error of $7.7\times 10^{-6}$ and an average of $1.9\times 10^{-6}$, well below the $10^{-5}$–$10^{-8}$ requirement for BFGS superlinear convergence [33]. The results are summarized in Table 9.

CB’s 300-evaluation budget reflects three compounding factors: dimensionality reduction (12 → 5), constraint elimination, and improved Hessian conditioning in lower dimensions.

### 3.8. Comparison with Derivative-Free Baselines

**Figure 8:**
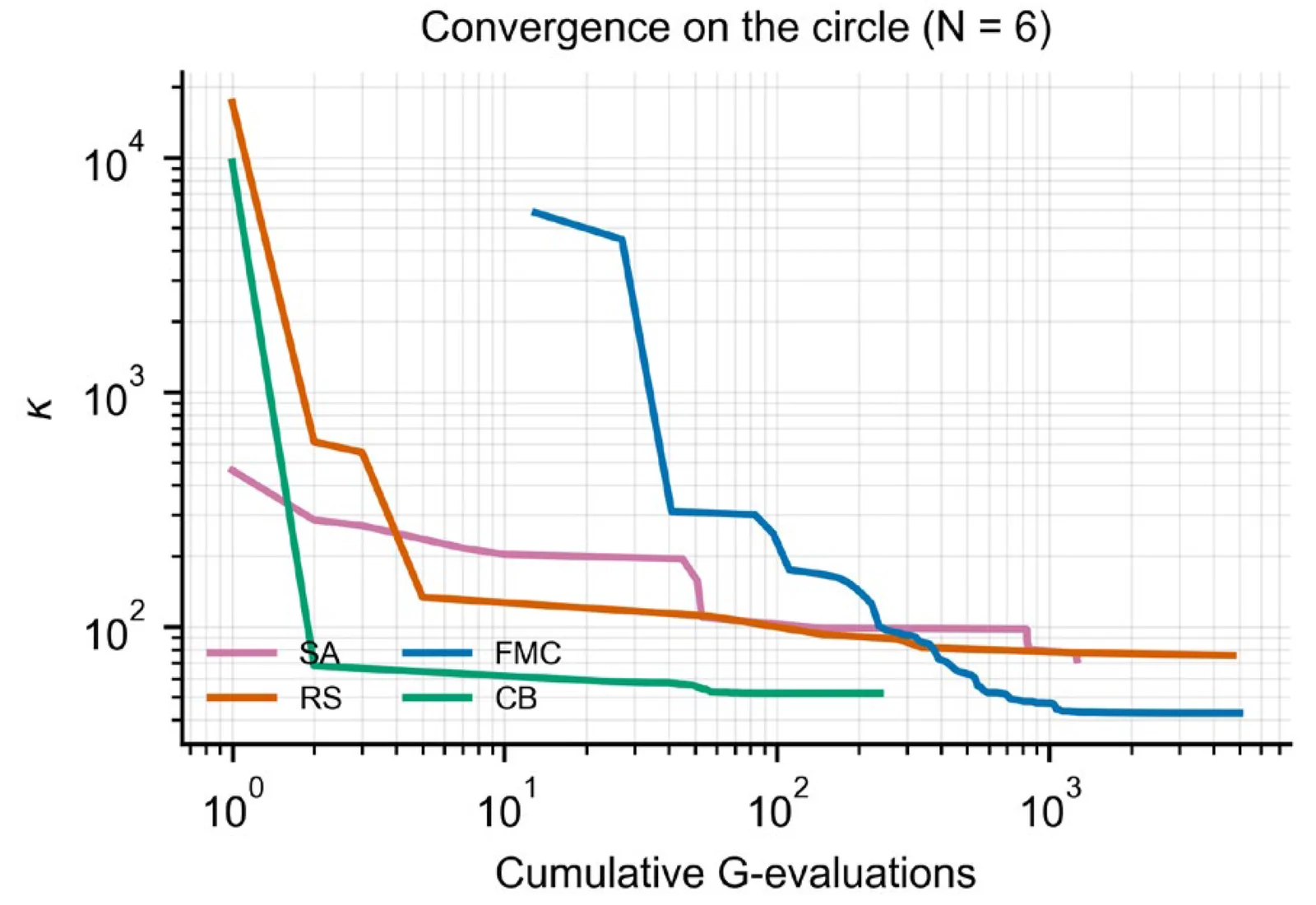
Convergence of the four methods on the circle (κ versus cumulative G-evaluations, log scale).

To situate the gradient-based methods against the derivative-free alternative, we compare simulated annealing (SA) and pure random search (RS) with the gradient methods on the circle (N = 6). Each derivative-free method is given the same 5000-evaluation budget as a single fmincon (FMC) run and repeated five times. The SA settings are initial temperature, T_0_ = 500; final temperature, T_min_ = 0.5; geometric cooling with factor 0.97; and 25 inner sweeps per temperature, where each sweep proposes a single-sensor Gaussian perturbation whose step size scales as 100·(T/500), with infeasible proposals projected back onto the circle. RS draws independent uniform samples over the circle and retains the best. The results are summarized in Table 10 and Figure 8.

At an equal evaluation budget, the gradient methods achieve a markedly lower condition number (FMC 43.8, CB 52.0 versus SA 69.7, RS 67.6), and CB attains a comparable result with only 192 evaluations (~26× fewer). This gap reflects a fundamental difference between the two families. Random search and simulated annealing require no derivative and handle constraints trivially, and their global exploration is a genuine advantage; however, each of their evaluations only returns a single value, with no information about where to move next, so most of the budget is spent probing rather than descending. A gradient step, in contrast, moves along the direction of steepest descent provided by the analytical gradient, so each evaluation contributes to convergence. The derivative-free methods therefore trade their convenience and global search for many more function evaluations to reach a comparable solution, whereas the gradient methods trade the cost of deriving the gradient for much faster convergence.

### 3.9. Minimum-Spacing Constraint Study

To verify that the framework accommodates an engineering minimum-spacing constraint, we add the pairwise constraints ‖p_i_ − p_j_‖ ≥ d_min_ to the FMC/SQP formulation on the circle (*N* = 6) and sweep d_min_ from 0 to 400 m. The results are summarized in Table 11.

The unconstrained optimum already separates the sensors well (minimum distance ≈ 305 m); the constraint is slack for d_min_ ≤ 200 m and becomes binding at d_min_ = 300 m, beyond which the optimum degrades only mildly, from κ ≈ 43.3 to 45.6 at d_min_ = 400 m (≈+5%). The same constraint can be imposed in BP as a barrier term and in CB as a forbidden-interval adjustment.

### 3.10. Synthetic Inversion Verification

**Figure 9:**
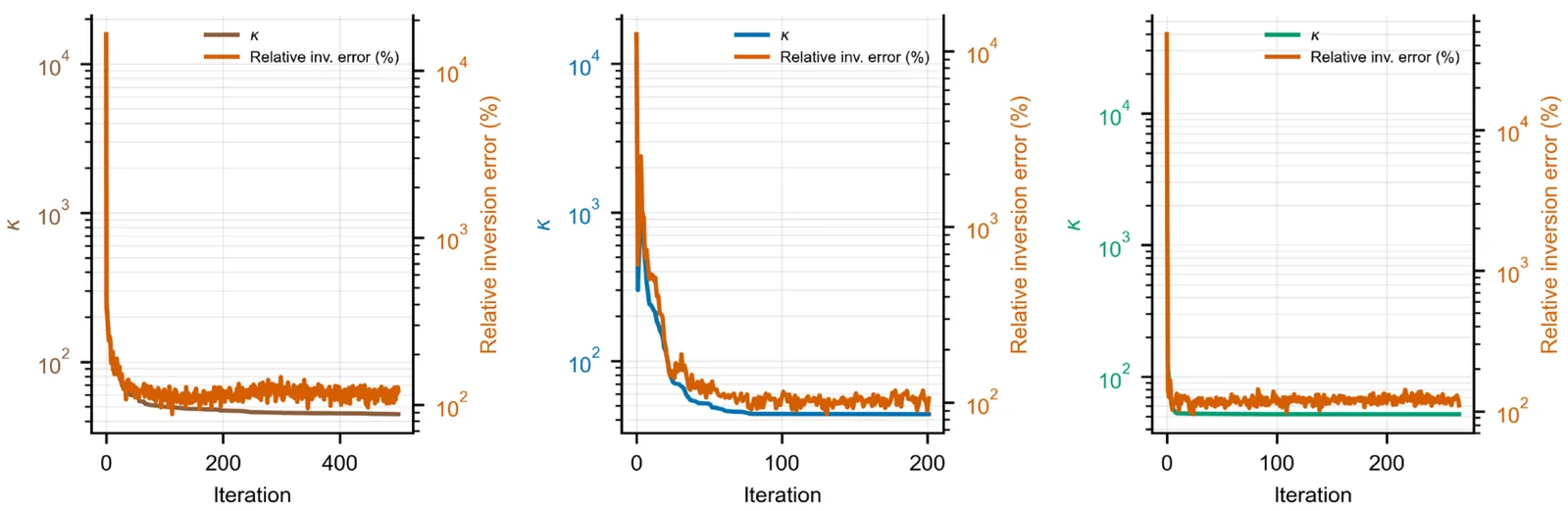
Synchronous decrease in condition number and inversion error.

To confirm that κ reduction translates to improved inversion accuracy, optimization paths are tracked from a random initial value ($\kappa_{0}\approx 1.5\times 10^{4}$) on a circular region. A double-couple source ($M={\left[0,1,0,0,0,-1\right]}^{T}$) is fixed and 10% Gaussian noise is added; least-squares inversion is performed over 50 repetitions at each intermediate configuration. Figure 9 presents the results.

The κ and error curves in Figure 9 are strongly coupled, reducing κ from approximately $1.5\times 10^{4}$ to approximately 50 reduces inversion error by approximately 50-fold. CB achieves inversion accuracy comparable to FMC despite higher final κ (52.0 vs. 43.8) because at κ≈50 the 10% noise level becomes the dominant error source—further κ reduction yields diminishing returns. This indicates that for practical deployments with 5–10% noise, optimizing κ below approximately 50 provides marginal additional benefit.

## 4. Limitations

This work is subject to several limitations that arise from the physical assumptions of the forward model and from the computational complexity inherent to the problem; these define the boundary of the conclusions that can be drawn.

Homogeneous isotropic medium: The forward model assumes far-field, homogeneous, isotropic conditions, and the analytical gradient derivation rests entirely on this assumption. In heterogeneous or anisotropic media, the P-wave radiation pattern and travel paths change, so the response matrix ***G*** is different, and the optimal configurations reported here cannot be transferred to such media without recomputing G through an appropriate Green’s function. The derivation framework itself remains structurally valid, but its quantitative results are specific to the assumed medium.

Source-position dependence: The condition number κ(***G***) is an intrinsic function of the source position: no single sensor configuration is optimal for all source positions, so a layout optimized at one depth is necessarily suboptimal at another. This is a property of the problem itself rather than an artifact of the experimental scope, and it means the reported condition numbers are representative of a source position, not universal. Accommodating source-location uncertainty would require reformulating the objective as a robust criterion.

Computational complexity of the gradient: Although the analytical gradient removes the 2*N* + 1 finite-difference evaluations, each gradient still requires a singular-value decomposition of the *N* × 6 response matrix, at a cost that grows as O(*N*^3^) in the number of sensors. The method is therefore inherently expensive for very large arrays, and this cost cannot be removed within an SVD-based formulation.

Combinatorial barrier of the center-boundary method: The center-boundary (CB) parameterization certifies global optimality of the discrete allocation only through exhaustive enumeration, whose count grows combinatorially as C(*N* + *P* − 1, *P* − 1); FMC/SQP likewise computes constraint gradients by finite differences at O(*NP*) forward perturbations per gradient. These are hard combinatorial and computational barriers inherent to the two formulations, so exact global optimality is attainable only for small instances, and larger instances are computationally prohibitive.

## 5. Conclusions

This paper derived the analytical gradient of the condition number κ(G) with respect to sensor positions for microseismic moment tensor inversion, by combining singular value perturbation theory with the chain rule of direction cosines. The gradient is obtained at negligible overhead beyond the SVD required for condition number evaluation, and its accuracy is verified to within $10^{-5}$ of the relative error. Based on this gradient, three complementary optimization strategies are established—BP (penalty function), FMC/SQP (explicit constraints), and CB (center-boundary parameterization)—that span the generality–efficiency spectrum of constraint handling. Systematic experiments demonstrate that gradient-based methods substantially outperform the existing OLD baseline in both accuracy and robustness, with the choice among the three methods governed by region geometry and computational budget. Synthetic inversion confirms that condition number reduction directly translates to inversion accuracy improvement.

The two-step gradient derivation framework—SVD perturbation followed by domain-specific forward-model differentiation—is transferable to sensor placement problems across structural health monitoring, geodetic network design, and optimal experimental design. Extensions to robust optimization under source location uncertainty, heterogeneous media, and large-scale hierarchical deployment represent natural next steps.

## Figures and Tables

**Table 1:** Summary of four methods used in numerical tests.

Method	Description
OLD	Original polar-coordinate random-direction coordinate descent [24]
BP	BFGS + quadratic penalty with analytical gradient
FMC	Sequential quadratic programming + analytical gradient
CB	Center-boundary parameterization + BFGS

**Table 2:** Direct comparison from the same initial configuration.

Shape	$\kappa_{0}$	Method	Final κ	Time (s)	G Evals	Iterations
Circle	799.0	OLD	799.4	0.036	768	32
		BP	42.9	0.072	1674	1000
		FMC	43.3	0.663	5000	344
		CB	52.0	0.012	187	85
Square	317.4	OLD	317.3	0.014	864	36
		BP	52.5	0.007	139	34
		FMC	37.1	0.089	5000	328
		CB	46.0	0.005	52	6
Triangle	521.9	OLD	522.0	0.013	840	35
		BP	231.6	0.004	60	8
		FMC	73.3	0.081	5005	331
		CB	Explodes (10^35^)	0.005	43	1
Hexagon	766.0	OLD	731.5	0.009	816	34
		BP	146.4	0.001	44	9
		FMC	54.9	0.073	5007	333
		CB	64.4	0.005	208	27
Octagon	1768.0	OLD	1727.2	0.010	840	35
		BP	172.7	0.003	65	13
		FMC	44.9	0.106	2536	155
		CB	54.8	0.009	353	172

**Table 3:** Statistical comparison on five simple region shapes.

Shape	OLD	BP	FMC	CB	OLD σ	BP σ	FMC σ	CB σ
Circle	51.2	43.6	43.6	52.3	21.7	0.8	1.5	1.2
Square	48.6	37.1	35.4	46.4	14.1	2.9	0.8	25.4
Triangle	109.6	371.7	71.5	—	33.9	309.4	1.1	—
Hexagon	64.1	120.6	53.7	60.5	22.1	66.2	1.4	4.9
Octagon	55.3	104.7	45.6	55.5	8.4	50.8	1.7	1.3
Average	65.8	135.5	50.0	53.7	20.0	86.0	1.3	8.2

— marks frequent line search failures; data unreliable. Averages exclude these shapes.

**Table 4:** Single-run comparison (one run per method and shape).

Shape	OLD Single Run (κ)	FMC Single Run (κ)
Circle	45.5	43.3
Square	49.2	34.4
Triangle	201.5	70.4
Hexagon	95.9	53.8
Octagon	58.7	46.6

**Table 5:** OLD with 100 random starts (global-search dimension).

Shape	OLD Best of 100 (κ)	OLD Top-75% Mean	Total G-Evals (100 Starts)
Circle	43.3	45.3	213,000
Square	35.3	38.1	238,152
Triangle	73.7	92.4	235,080
Hexagon	53.3	57.1	215,568
Octagon	45.4	49.7	214,656

**Table 6:** Comparison on complex regions.

Region	OLD	FMC	CB
Annulus ($R_{\mathrm{in}}=200$, $R_{\mathrm{out}}=500$)	45.8	43.5	74.6
Disconnected patches (2 circles, R=250)	147	71.0	79.7

**Table 7:** Computational scaling of the analytical gradient versus the finite-difference gradient.

N	G-Evals (Analytical)	G-Evals (Finite Difference)	Analytical Gradient Time	Finite-Difference Gradient Time	Speedup
6	1	13	2.7 × 10^−5^ s	1.7 × 10^−4^ s	6.3×
20	1	41	3.2 × 10^−5^ s	5.9 × 10^−4^ s	18.2×
50	1	101	6.4 × 10^−5^ s	1.8 × 10^−3^ s	28.6×
100	1	201	1.2 × 10^−4^ s	3.5 × 10^−3^ s	30.2×

**Table 8:** Analytical vs. finite-difference gradients (circle, five trials).

Method	κ	$\sigma_{\kappa }$	Avg. *G* Evals	Wall Time (s)
OLD	47.5	11.9	2328	0.04
FDG	43.5	0.6	15,624	0.37
BP	43.3	0.5	1496	0.07
FMC	43.6	1.4	5007	0.66
CB	52.0	0.0004	250	0.01

**Table 9:** Comparison of computational cost per iteration and per full optimization.

Cost Component	OLD	BP	FMC	CB
G evaluations/iteration	24 (fixed)	1–3	1–15	1–3
Gradient source	None	Analytical	Analytical	Analytical
Constraint handling	Projection	Penalty gradient	SQP linearization	Embedded in param.
Hessian update	None	$O\left({\left(2N\right)}^{2}\right)$	$O\left({\left(2N\right)}^{2}\right)$	$O\left({\left(N-1\right)}^{2}\right)$
Full optimization G evals	~2300	~750–1500	~5000	~300

**Table 10:** Derivative-free baselines versus the gradient-based methods on the circle (equal 5000-evaluation budget).

Method	Final κ (Mean ± Std)	G-Evaluations
Random search (derivative-free)	67.6 ± 3.6	5000
Simulated annealing (derivative-free)	69.7 ± 5.5	5000
FMC/SQP (analytical gradient)	43.8	5000
CB (analytical gradient)	52.0	192

**Table 11:** Minimum-spacing constraint study on the circle (*N* = 6).

d_min_ (m)	Final κ	Achieved Minimum Inter-Sensor Distance (m)
0	43.26	305.4
100	42.84	288.9
200	43.15	301.9
300	42.87	300.0
400	45.57	400.0

## Data Availability

The data presented in this study are available on request from the corresponding author due to privacy.
